# Three-Dimensional Soft Tissue Reconstructive Planning in Orthoplastic Oncology: A Systematic Review

**DOI:** 10.7759/cureus.113884

**Published:** 2026-08-03

**Authors:** Alexander F Dagi, Nadeem Jones, Ying Yu, Wakenda Tyler, Jarrod T Bogue

**Affiliations:** 1 Division of Plastic Surgery, Columbia University, New York, USA; 2 Department of Orthopedic Surgery, Columbia University, New York, USA

**Keywords:** orthopedic oncology, patient-specific instrumentation, soft tissue reconstruction, surgical planning, three-dimensional printing

## Abstract

Complex orthopedic oncology cases require coordinated planning between tumor surgeons and reconstructive plastic surgeons, yet a technological disconnect exists. While orthopedic oncologists seek to achieve millimeter-level precision using three-dimensional-guided systems for bone tumor resection from computed tomography scan reformats, plastic surgeons rely on traditional two-dimensional imaging for reconstructive planning in the same cases. We conducted a systematic review of reviews examining three-dimensional technologies in orthopedic oncology and their relevance to soft tissue reconstructive planning. We searched PubMed, Embase, and Cochrane databases through November 2024, using terms combining "three-dimensional", "orthopedic oncology", and "bone tumor". Two independent reviewers performed dual screening with disagreements resolved by consensus. After reviewing 325 articles, we identified 16 reviews (three systematic, 13 narrative) published between 2009 and 2024. Despite comprehensive evaluation across these reviews encompassing hundreds of primary studies, no applications for soft tissue reconstruction planning were identified. Orthopedic teams demonstrated remarkable progress in precision with patient-specific instrumentation achieving superior negative margin rates (89% versus 68% for conventional methods) and cutting deviations of 0.3-4 mm, yet the same imaging data used for skeletal planning, containing nearly all information necessary for soft tissue reconstruction, remained unutilized for reconstructive purposes. Three-dimensional planning technologies have transformed orthopedic oncology but are typically absent from soft tissue reconstruction planning in the same cases. A significant opportunity exists for plastic surgeons to integrate into existing three-dimensional workflows through defect volume analysis, vessel mapping, flap design optimization, and coordinated reconstructive timing within navigation-guided resections.

## Introduction and background

Complex orthopedic oncology cases increasingly require coordinated planning between tumor surgeons and reconstructive plastic surgeons to optimize both oncologic and functional outcomes [1,2]. Yet despite this recognized need for multidisciplinary collaboration, a technological disconnect has emerged. While orthopedic oncologists aim for sub-millimeter precision using patient-specific instrumentation (PSI) and navigation systems for bone tumor resection [3], plastic surgeons rely on intuition and judgment from preoperative and intraoperative assessment of two-dimensional (2D) imaging and three-dimensional (3D) reconstructions without formal modeling of the planned soft tissue reconstruction. This gap is particularly striking given that complex sarcoma resections invariably create composite defects involving both skeletal and soft tissue components, yet current 3D workflows address only the skeletal planning component.

Our recent systematic review of plastic surgery literature examining 3D planning applications in lower extremity soft tissue sarcoma reconstruction found no published evidence for such applications [1]. This complete absence of soft tissue integration is remarkable given the complexity of volumetric defects inherent to oncologic resections and the well-established technical capabilities of 3D planning systems. To understand this disconnect and identify opportunities for integration, we conducted a systematic review of reviews to examine how 3D planning technologies are currently applied in orthopedic oncology and what barriers prevent their adoption for soft tissue reconstruction.

The potential applications of 3D technologies in soft tissue reconstruction are substantial. Volumetric planning enables the prediction of post-resection defects, optimization of flap design based on precise defect geometry, recipient vessel mapping, and coordinated timing of reconstructive interventions within navigation-guided resections. Early plastic surgery involvement in 3D planning processes could ensure reconstructive considerations are integrated alongside oncologic objectives. Understanding the barriers to adoption and developing implementation strategies aligned with surgical innovation frameworks are essential for advancing truly integrated orthoplastic care.

## Review

Search strategy

**Figure 1:**
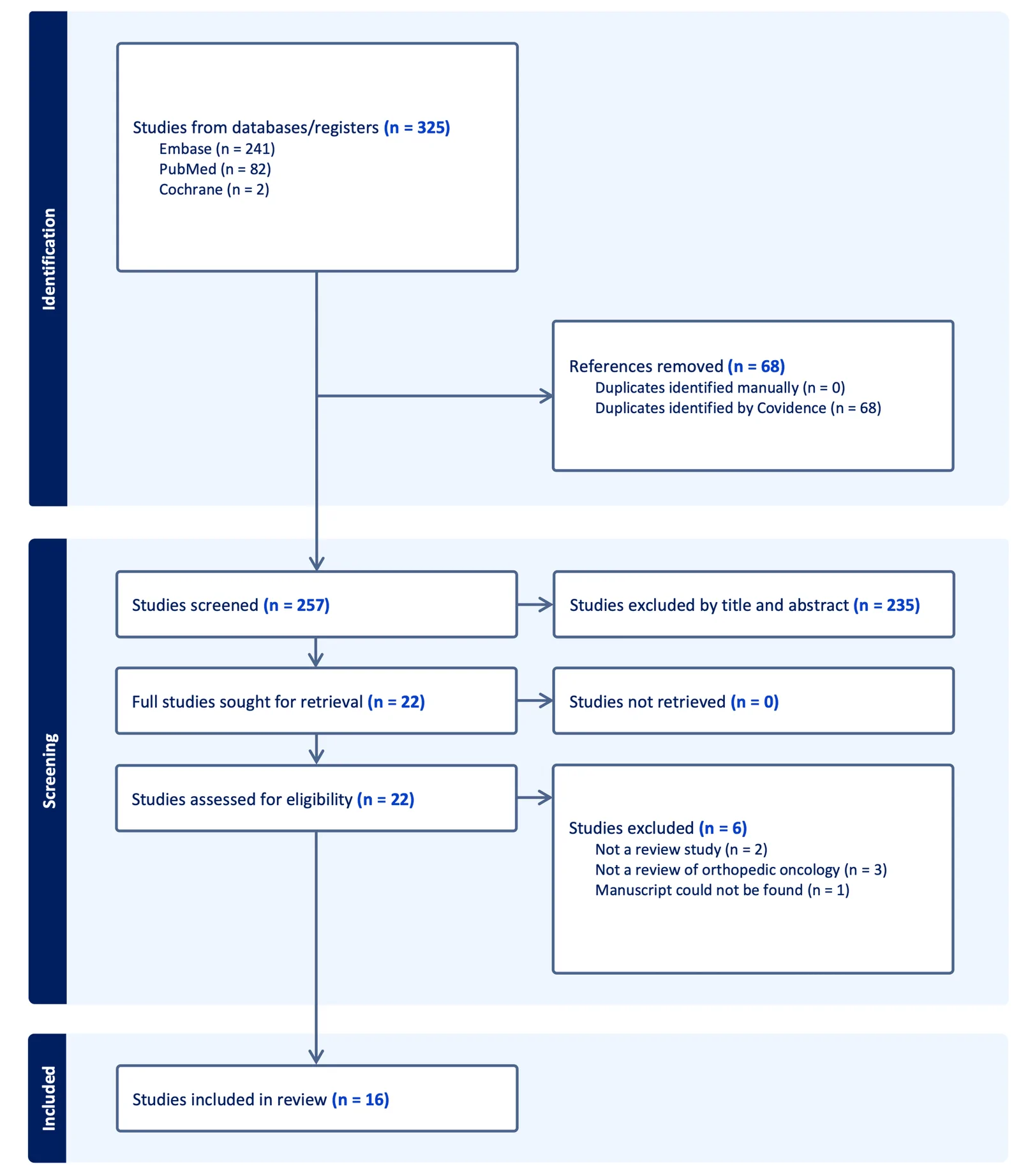
PRISMA chart for 3D planning in orthopedic oncology review study selection Screening completed according to PRISMA guidelines [4]. PRISMA: Preferred Reporting Items for Systematic Reviews and Meta-Analyses

This systematic review of reviews was conducted following the Preferred Reporting Items for Systematic Reviews and Meta-Analyses (PRISMA) 2020 guidelines [4]. A systematic literature search was conducted across three electronic databases (PubMed, Embase, and Cochrane) from database inception through November 2024 using terms combining "three-dimensional", "orthopedic oncology", and "bone tumor". All search results were imported into Covidence (Veritas Health Innovation, Melbourne, Australia) for systematic screening and data extraction (Figure 1).

A review of reviews methodology was chosen because the literature on 3D technology use in orthopedic oncology is prolific, with numerous published reviews. Individual primary studies vary significantly in methodology, technology platforms, anatomical sites, and outcome measures, making direct comparison challenging. By synthesizing existing reviews, we captured the collective understanding of 3D technologies across multiple subspecialties while identifying consensus opinions and knowledge gaps relevant to plastic surgical applications.

Study selection followed a two-stage process with predetermined eligibility criteria. Two independent reviewers (N.E.J. and Y.Y.) screened at each stage using dual-review methodology, with disagreements resolved through discussion with a third author (A.F.D.). Articles were initially screened by title and abstract and then by full text. Studies were included if they were review articles (systematic reviews, meta-analyses, narrative reviews, or scoping reviews) that focused on 3D technology applications in orthopedic oncology (including planning software, printing, cutting guides, or navigation systems), were published after 2009, and had English full-text manuscripts available.

Full-text screening was conducted with the same dual-review process. Articles were excluded if they were primary research studies, conference abstracts, letters, or commentaries. Additionally, reviews that only tangentially mentioned 3D applications in orthopedic oncology without substantial discussion were excluded. The screening process was documented using a PRISMA flow diagram.

Quality assessment of the included reviews was conducted using a hybrid approach combining elements from the Measurement Tool to Assess Systematic Reviews 2 (AMSTAR-2) and the Scale for the Assessment of Narrative Review Articles (SANRA) [5,6]. This combined approach was chosen to appropriately evaluate both systematic and narrative reviews, accounting for the different methodological expectations of each review type. The six evaluated quality domains were as follows: (1) clear statement of objectives, (2) comprehensive literature search strategy, (3) quality of evidence synthesis, (4) clinical applicability of conclusions, (5) technological assessment methodology, and (6) discussion of limitations and future directions. Each domain was scored 0 (no evaluation), 1 (partial evaluation), or 2 (complete evaluation).

Data extraction specifically focused on identifying opportunities for plastic surgery integration, soft tissue considerations, and reconstructive applications. A standardized extraction form captured the study characteristics (author, year, country, journal), review type, focus on oncology, technologies evaluated, anatomical sites covered, surgical process stages addressed, clinical outcomes reported (resection margins, functional outcomes, complications, cost analyses), and technical characteristics.

Given the anticipated heterogeneity in review types and focus areas, a narrative synthesis approach was chosen. Data were synthesized with a specific focus on information relevant to plastic surgical collaboration in 3D planning workflows. Special attention was paid to identifying indications and opportunities for plastic surgery involvement in the 3D planning process.

Results

The primary search strategy yielded 325 articles (Figure 1). After the removal of duplicates, 257 unique records were identified across the three databases. Screening of titles and abstracts excluded 241 records, leaving 16 reviews eligible for full-text analysis. These reviews were published between 2009 and 2024, with a notable temporal trend toward increased publication in recent years.

Of the included reviews, three were systematic reviews [3,7,8], and 13 were narrative reviews [9-21] (Table 1). Geographic distribution showed representation from Asia, Europe, and North America, with notable contributions from Italy, China, Poland, and the United States. The included reviews examined a range of 3D technologies with applications in orthopedic oncology, including PSI and cutting guides, navigation systems, 3D-printed implants and prostheses, and planning software platforms. The pelvis was the most frequently discussed anatomical site across reviews, followed by the femur and tibia.

**Table 1:** Orthopedic oncology review articles queried for soft tissue reconstruction 3D planning *Quality scores based on hybrid AMSTAR-2/SANRA assessment. Systematic reviews evaluated on six domains (clear objectives, comprehensive literature search, quality of evidence synthesis, clinical applicability, technological assessment methodology, discussion of limitations) with a maximum score of 12 points. Narrative reviews assessed using the same criteria with adjusted expectations for the literature search strategy. PSI: patient-specific instrumentation; AMSTAR-2: Measurement Tool to Assess Systematic Reviews 2; SANRA: Scale for the Assessment of Narrative Review Articles

Authors	Year	Journal	Anatomical focus	Primary focus	Quality score*	Reference
Systematic reviews						
Bruschi et al.	2023	J Bone Oncol	Long bones, pelvis	PSI vs navigation	12	[3]
Yen et al.	2021	J Long Term Eff Med Implants	Pelvis, femur, tibia, spine	3D printing in musculoskeletal oncology	12	[7]
Fan et al.	2023	Phys Med Biol	Pelvis, long bones	Image-guided orthopedic surgery	8	[8]
Narrative reviews						
Qu et al.	2024	Int J Surg	Pelvis, femur, foot/ankle	3D printed bone prosthesis materials	6	[9]
Park and Kang	2022	Clin Exp Pediatr	Pelvis, femur, tibia	3D printing for bone tumors	8	[10]
Kotrych et al.	2023	EFORT Open Rev	Pelvis, femur, tibia, spine	Patient-specific implants in oncology	7	[11]
Cartiaux et al.	2011	Eur Musculoskelet Rev	Pelvis	Computer-assisted pelvic tumor surgery	9	[12]
Wong and Kumta	2014	Curr Surg Rep	Pelvis, femur, tibia	Computer navigation in orthopedic oncology	8	[13]
Fehlberg et al.	2009	Recent Results Cancer Res	Pelvis	Computer-assisted pelvic tumor resection	9	[14]
Angelini et al.	2020	Int Orthop	Pelvis, femur, tibia, foot/ankle, upper extremity	3D-printed custom prostheses design	6	[15]
Spalek et al.	2024	Chin Clin Oncol	Pelvis, femur, foot/ankle, upper extremity	3D printing challenges in oncology	7	[16]
Satcher	2013	Orthop Clin North Am	Pelvis, femur, tibia, foot/ankle, upper extremity	Intraoperative navigation in tumor surgery	9	[17]
Benady et al.	2022	J 3D Print Med	Pelvis, femur, tibia	3D printing in orthopedic oncology	10	[18]
Aiba et al.	2023	Curr Oncol	Pelvis, femur, tibia, upper extremity	Patient-specific cutting guides	9	[19]
Wong et al.	2018	Adv Exp Med Biol	Pelvis, femur, tibia	Computer navigation in tumor surgery	7	[20]
Bird	2014	Curr Oncol Rep	Pelvis, femur, tibia	General uses of 3D planning in orthopedic oncology	7	[21]

Quality assessment of the included reviews demonstrated variable methodological rigor (Table 1). Systematic reviews generally scored higher on methodology assessments, with a mean total quality score of 11.7±0.6 compared to narrative reviews at 8.2±1.1 (p<0.001). The most significant differences between review types appeared in comprehensive literature search strategy (systematic reviews 1.7±0.5 versus narrative reviews 0.4±0.5; p<0.001) and quality of evidence synthesis (systematic reviews 2.0±0 versus narrative reviews 1.2±0.4; p<0.001).

Absence of Soft Tissue Reconstruction Applications

Despite comprehensive evaluation of 3D technologies across 16 reviews encompassing hundreds of primary studies in orthopedic oncology, no applications for soft tissue reconstruction planning were identified. This represents a complete technological disconnect between the sophisticated skeletal planning capabilities now routinely available and the reconstructive requirements of the same patients.

Critical reconstructive considerations that remained unaddressed across all reviewed studies include the following: (1) soft tissue defect prediction based on virtual tumor resection, (2) recipient vessel relationship mapping for flap perfusion planning, (3) flap design optimization based on precise defect volume and geometry, (4) donor site planning accounting for defect dimensions, and (5) integration of reconstructive timing into surgical workflows. Notably, no reviewed studies addressed the interface between custom implants and overlying soft tissue coverage, despite this being a critical determinant of reconstructive success and a frequent source of complications.

Technologies evaluated across reviews included PSI and cutting guides, navigation systems (with computed tomography and magnetic resonance fusion), 3D-printed implants and prostheses, and planning software platforms (Mimics®, OrthoView®, SurgiCase® (Materialise, Leuven, Belgium)). Table 2 provides a summary of these technologies, their key applications, and the reviews that discussed them in detail.

**Table 2:** Precision capabilities versus potential applications MSTS: Musculoskeletal Tumor Society

Technology	Skeletal applications	Achievements	Soft tissue applications	Opportunities	References
Patient-specific instrumentation	Cutting guides, tumor marking	0.3-4 mm deviation	None identified	Flap sizing, defect analysis	[3,10,15,18,19,21]
Navigation systems	Real-time surgical tracking	89% negative margins	None identified	Vessel mapping, recipient site planning	[3,8,13,14,17,20]
3D-printed implants	Custom prostheses	MSTS scores 28.3 vs 26.2	None identified	Implant-tissue interface planning	[7,9-11,15,16,18]
Planning software	Virtual resection	Sub-2 mm accuracy	None identified	Volumetric reconstruction planning	[8,12,14,20]

Precision in Skeletal Applications

Orthopedic teams achieve remarkable precision using 3D technologies. PSI demonstrated superior negative margin rates (89% versus 68% for conventional methods) and cutting deviations of 0.3-4 mm across multiple studies [2]. Navigation systems reduced tumor cut-through rates from 20.7% to 6.9% [3]. Functional outcomes improved significantly, with Musculoskeletal Tumor Society (MSTS) scores of 28.3 versus 26.2 for conventional techniques (p=0.037) [2]. Intralesional resection rates improved dramatically when using computer-assisted tumor surgery techniques for pelvic and sacral tumors, with rates reduced from 29% to 8.7% [4]. These improvements translate to clinically meaningful changes in local recurrence rates, with some series demonstrating reductions from 70% (using traditional surgical techniques) to 13-25% (using navigation-guided approaches) [4].

Clinical Outcomes Across 3D Technology Applications

PSI demonstrated superior precision in achieving negative margins compared to conventional methods. A retrospective study of 19 patients with pelvic tumors showed improved negative margin rates in the PSI group (89%) versus the control group (68%) [19]. Navigation systems reduced the risk of tumor cut-through from 20.7% to 6.9%, significantly improving outcomes for bone tumor resections [8]. Both PSI and navigation systems demonstrated the effective provision of high accuracy for long bone resections, with cutting deviations ranging from 0.3 mm to 4 mm, with the majority reporting mean errors of less than 2 mm [3].

Postoperative function, measured using the MSTS score, was available from limited studies and demonstrated improvements with PSI in bone tumor resection around knee joints. One study reported mean MSTS scores of 28.3 for the PSI group versus 26.2 for the control group (p=0.037) [19]. Non-pelvic reconstructions demonstrated better functional outcomes compared to pelvic procedures, attributed to simpler biomechanical demands and faster recovery times. Better preservation of native joints was noted in PSI-guided procedures.

Overall complication rates for surgeries using 3D technologies were comparable to those of conventional techniques, with no significant increase in adverse events [17]. PSI typically required 2-4 weeks for preoperative preparation [3,19], while navigation systems required 15-65 minutes for intraoperative setup [20]. Multiple studies noted that navigation technology was generally more time-consuming intraoperatively compared to PSI, though operative time varied across studies influenced by surgical complexity and team expertise [21].

Discussion

This systematic review of reviews reveals an important disparity: 3D planning technologies have demonstrated clear benefits for skeletal resection and reconstruction in orthopedic oncology, yet their application to soft tissue reconstruction in the same patients remains completely undeveloped. Our review identified 16 substantial reviews encompassing hundreds of primary studies, none of which addressed soft tissue defect prediction, vessel mapping, flap design optimization, or integration of reconstructive timing into 3D planning workflows.

The technical foundation for 3D soft tissue planning integration is well-established. PSI achieves cutting deviations of 0.3-4 mm, with most studies reporting less than 2 mm [3]. Meta-analyses of patient-specific surgical guides across multiple anatomical sites demonstrate mean angular deviations of 1.8-3.5 degrees, with accuracy in 90.9% of cases meeting <5-degree error thresholds [22]. Navigation systems utilizing computed tomography-magnetic resonance fusion achieve similar precision for complex anatomical regions. Quality assurance frameworks from the Radiological Society of North America (RSNA) and International Organization for Standardization (ISO 13485, ISO 10993) provide validated protocols for ensuring dimensional accuracy and biocompatibility throughout the design and manufacturing process [23].

The same segmentation, virtual resection planning, and 3D modeling tools used for skeletal reconstruction are directly applicable to soft tissue analysis. Volumetric defect prediction can be calculated from virtual tumor resection models. Recipient vessel anatomy can be identified from preoperative imaging during surgical planning. Flap geometry can be optimized to match precise defect dimensions. Technical barriers to implementation are minimal; the limitations are organizational, educational, and economic.

Several barriers impede 3D technology adoption in soft tissue reconstruction. At present, there is inadequate reimbursement for 3D planning and printing services. Current Procedural Terminology (CPT) codes for 3D printing are categorized as Category III (temporary/emerging technology), meaning they are rarely reimbursed by insurance or carry substantially reduced reimbursement compared to conventional planning [24]. This creates a fundamental economic problem: 3D planning requires upfront investment (software $50,000-$250,000, printers $100,000-$500,000, personnel training and time), requires 2-4 weeks of production time for custom guides, and yet generates no direct revenue stream when services cannot be billed [24,25]. One single-site economic analysis estimated that a minimum of 63 3D models or surgical guides must be produced annually (1.2 per week) simply to recover fixed equipment costs, assuming operating room savings of $62 per minute [26]. For reconstructive cases where indirect benefits (vessel mapping, defect prediction) may not translate to measurable operative time savings, this breakeven threshold becomes prohibitive. A systematic review of 227 surgical 3D printing publications found cost-effectiveness explicitly supported in only 7% of studies, with quantitative economic data almost entirely absent [27]. Without robust reimbursement or institutional commitment to absorb costs, plastic surgeons cannot justify adopting 3D planning technologies.

Implementation of 3D planning requires reorganization of established surgical workflows. A qualitative study of 3D printing implementation in Swedish healthcare identified organizational barriers (33 mentions) as exceeding technical barriers, including lack of central hospital-level decision support (six mentions), inadequate premises and equipment infrastructure (four mentions), requirements for specialized technical competence (four mentions), and cultural resistance from conservative surgical staff (four mentions) [28]. Introduction of 3D planning into orthoplastic workflows requires coordination among orthopedic surgeons, plastic surgeons, radiologists, biomedical engineers, and clinical staff, a level of multidisciplinary coordination that currently exists only in a few specialized centers.

Regulatory uncertainty surrounding 3D-printed devices remains a substantial barrier. The Food and Drug Administration's regulatory framework for 3D-printed devices remains incomplete, with particular ambiguity regarding customized instruments and implants [29]. Customization creates challenges for design control, quality assurance, and manufacturing validation. Sterilization requirements, biocompatibility testing (ISO 10993), and material validation for PSI require technical expertise.

Finally, plastic surgeons and orthopedic surgeons traditionally train separately, operate in different service lines, and may not have developed collaborative relationships necessary for integrated 3D planning. Early plastic surgery involvement in sarcoma planning remains the exception rather than the standard, despite evidence that orthoplastic collaboration improves functional outcomes and reduces complications [30-33].

To help close this gap, the Idea, Development, Exploration, Assessment, Long-term Study (IDEAL) framework, a staged, validated model for introducing surgical innovations, along with its device-specific extension (IDEAL-D), offers a practical pathway for moving 3D soft tissue planning from concept to standard practice [34]. Applied here, the first stage would involve developing a method to automatically calculate soft tissue defect volume from existing virtual tumor resection models and validating vessel-mapping algorithms against actual intraoperative anatomy. Success at this stage would be defined by concrete accuracy benchmarks, for example, defect volume calculations within 5 mm and vessel location predictions within 2 mm of ground truth. Achieving this will require close collaboration between plastic surgeons, radiologists, and biomedical engineers to establish technical feasibility before any clinical use.

Strengths and Limitations

This review focused on review articles rather than primary studies, which limited the depth of analysis regarding specific surgical outcomes and technical innovations. While this approach provided broad synthesis of existing literature and identified consensus gaps, it may have excluded emerging experimental uses of 3D technology not yet captured in reviews. Review articles by definition are published several years after primary studies, potentially missing very recent innovations in soft tissue planning.

Publication bias remains a concern, as studies demonstrating positive outcomes are disproportionately represented in the literature. Negative or inconclusive findings regarding 3D technology benefits are less likely to be published, potentially leading to the overestimation of clinical efficacy or underestimation of trials of 3D technology in plastic surgery workflows. The heterogeneity of included reviews posed challenges in quality assessment, as systematic reviews adhered to rigorous methodologies while narrative reviews often lacked comprehensive search strategies and standardized evidence synthesis. Despite these limitations, our findings align with what is known about adoption barriers for surgical technologies generally and identify specific opportunities for plastic surgery integration that remain unexplored.

## Conclusions

3D planning technologies have become increasingly utilized in orthopedic oncology, with significant benefits for surgical precision, functional outcomes, and multidisciplinary collaboration. PSI and navigation systems have consistently demonstrated superior accuracy in achieving negative tumor margins, with cutting deviations typically less than 2 mm. These technologies have shown particular utility in complex anatomical regions such as the pelvis, where precision is critical for both oncologic and functional outcomes.

Despite these advances in skeletal planning and reconstruction, the integration of soft tissue reconstructive planning within 3D orthopedic oncology workflows remains completely undeveloped. The current literature focuses predominantly on skeletal applications, with minimal attention to critical reconstructive considerations such as soft tissue defect prediction, vessel mapping, and flap design optimization. This represents a significant missed opportunity for plastic surgeons to contribute expertise to multidisciplinary planning teams. The imaging data and software tools necessary for soft tissue planning integration already exist. The evidence supporting orthoplastic collaboration in limb salvage surgery is compelling; multidisciplinary approaches reduce amputation rates, decrease infectious complications, and improve functional outcomes. Early plastic surgery involvement in 3D planning could extend these benefits by enabling more precise preoperative reconstructive planning.
